# Thiourea, a ROS Scavenger, Regulates Source-to-Sink Relationship to Enhance Crop Yield and Oil Content in *Brassica juncea* (L.)

**DOI:** 10.1371/journal.pone.0073921

**Published:** 2013-09-18

**Authors:** Manish Pandey, Ashish Kumar Srivastava, Stanislaus Francis D'Souza, Suprasanna Penna

**Affiliations:** Nuclear Agriculture and Biotechnology Division, Bhabha Atomic Research Centre, Mumbai, India; National Taiwan University, Taiwan

## Abstract

In the present agricultural scenario, the major thrust is to increase crop productivity so as to ensure sustainability. In an earlier study, foliar application of thiourea (TU; a non physiological thiol based ROS scavenger) has been demonstrated to enhance the stress tolerance and yield of different crops under field condition. Towards this endeavor, present work deals with the effect of TU on photosynthetic efficiency and source-to-sink relationship of Indian mustard (*Brassica juncea*) for understanding its mode of action. The application of TU increased the efficiency of both PSI and PSII photosystems and vegetative growth of plant. The comparative analysis of sucrose to starch ratio and expression level of sugar transporters confirmed the higher source and sink strength in response to TU treatment. The biochemical evidence in support of this was derived from higher activities of sucrose phosphate synthase and fructose-1,6-bis-phosphatase at source; and sucrose synthase and different classes of invertases at both source and sink. This indicated an overall increase in photoassimilate level at sink. An additional contribution through pod photosynthesis was confirmed through the analysis of phosphoenol pyruvate carboxylase enzyme activity and level of organic acids. The increased photoassimilate level was also co-ordinated with acetyl coA carboxylase mediated oil biosynthesis. All these changes were ultimately reflected in the form of 10 and 20% increase in total yield and oil content, respectively under TU treatment as compared to control. Additionally, no change was observed in oil composition of seeds derived from TU treated plants. The study thus signifies the co-ordinated regulation of key steps of photosynthesis and source-to-sink relationship through the external application of TU resulting in increased crop yield and oil content.

## Introduction

The continuous increase in global population along with the growing urbanization and impending climate change imposes significant pressure for increasing agricultural crop productivity. At present, the total land used for agriculture is close to the sustainable limit of 15% of the Earth's surface that can be exploited for crop production [1]. Thus, researchers worldwide are exploring innovative methods for increasing crop yield on limited land resources [2]. The crop yield is a complex trait and is found to be dependent upon three interdependent factors such as generation of photosynthetic reductant, its assimilation into the carbon product and then translocation in different plant parts [3]–[5]. In *Brassica juncea*, since leaves are the prime site for photosynthesis, they are termed as “source” for the generation of photoassimilatory products mainly sucrose. Similarly, maturing seeds are mainly responsible for driving sucrose transportation away from leaves and hence termed “sink”. The sucrose translocation from source-to-sink is considered as the rate-limiting step for regulating the level of photoassimilate in sink and finally the crop yield [6], [7]. In source, there exists a continuous equilibrium between sucrose synthesis and its sequestration inside vacuole and/or transportation towards sink. Any disruption in this equilibrium leads to a higher accumulation of sucrose that mediates feedback inhibition of its synthesis. This ensures the activation of starch biosynthesis process and beyond certain threshold; leaf no longer behaves as source. Thus, in order to maintain higher strength and longevity of source, it is necessary to either avoid or delay the synthesis of starch in leaves. Apart from this, the sink strength is also an important determinant of sucrose translocation. The higher conversion rate of sucrose into either starch or oil increases the sink strength that supports the accumulation of more photoassimilates required for its growth. Thus, enhanced source and sink strength is a desirable trait that most of the plant biologists wish to achieve so as to enhance plant's harvest index and crop yield [5], [7].

**Table 1:** Quantitative parameters of growth in control and thiourea treated plants.

		Control	Thiourea
**Vegetative phase**	Average shoot length (cm)	7.3±0.5	17*±2.7
	Average leaf area (mm^2^)	5196±920	7629*±453
**Maturity phase**	Internodal distance between two pods (cm)	1.5±0.07	1.8*±0.07
	Pod length (cm)	4.3±0.05	4.5*±0.05
	Pod width (cm)	0.3±0.01	0.4*±0.01
	Average weight of 1000 seeds (g)	3.0±0.002	3.1*±0.001
	Average number of seeds per pod	12.7±0.33	14.1*±0.35

At vegetative phase, values represent mean ± SD of ten individual plants. At maturity phase, values represent mean ± SD of ten independent biological replicates where each replicate denotes the data pooled from ten plants. The significance of mean difference (P<0.05) was evaluated on the basis of student t-test and marked with asterisk (*).

The generation of photoassimilates occurs in chloroplasts through the process of photosynthesis in which light energy is utilized to convert gaseous CO_2_ into triose phosphate (Gly-3-P; glyceraldehyde-3-phosphate). The triose phosphates can either be transported into cytosol by the inner chloroplast membrane localized triose-phosphate transporter (TPT) to mainly feed into sucrose synthesis, or can be retained within the chloroplast for starch synthesis. The TPT activity is responsible for the high assimilation capability of source leaves [8]. In cytosol, the Gly-3-P gets converted into fructose-1,6-bis-phosphate (F-B-P) by the combined action of triose phosphate isomerase and aldolase and then initiates two-step sucrose biosysnthesis process catalyzed by fructose-1,6-bis-phosphatase (FBPase) and sucrose phosphate synthase (SPS). FBPase represents the first step and converts F-B-P into fructose-6-phosphaste (F-6-P). SPS catalyzes the second and rate-limiting step in which F-6-P and UDP-glucose are combined to make sucrose-6-phosphate, which is subsequently hydrolyzed to sucrose by sucrose phosphatase [9]. The SPS activity is regulated at different levels. The enzyme itself is regulated via allosteric activation by glucose-6-phosphate (G-6-P) and inhibition by inorganic phosphate. In addition, SPS is activated by light through changes in the phosphorylation state of several serine residues in the protein. Besides, when excess sucrose get accumulated during day time, feedback control also inhibits the activity of SPS that results in the accumulation of phosphorylated intermediates (F-B-P and F-6-P) and depletion of Pi [10]. This activates the starch biosynthesis pathway. Initially, F-6-P gets converted into G-6-P through isomerase activity. The G-6-P gets transported into chloroplast through G-6-P-transporter (G6PT) where the starch biosynthesis takes place through the rate limiting action of ADP-glucose pyrophosphorylase (AGPase [11]). In order to maintain low level of sucrose in leaves, plant possesses a set of sucrose transporters (SUTs) that mediate the transport of sucrose from source to sink [12]. In *Arabidopsis thaliana* genome, there are nine putative SUTs. AtSUC1, 2, 3, 8 and 9 represent high-affinity transporters while AtSUT4 is a low affinity tonoplast transporter. AtSUC5 performs the dual function to transport vitamin H and sucrose and AtSUC6 and 7 are pseudogenes. After unloading in sink, sucrose needs to be degraded to prevent any feedback inhibition on photosynthesis and to sustain transport from source-to-sink. The degration of sucrose is carried out by either invertases (INVs) that hydrolyze the sucrose into glucose and fructose or sucrose synthase (SuSy) that degrades sucrose into UDP-glucose and fructose. On the basis their localization at cell wall, cytoplasm and vacuole, INVs are termed as cwINVs, cINVs and vINVs, respectively [13]. The INVs and SuSy also function to lower the sucrose level in leaves hence are important for maintaining the source strength during day time. After reaching the sink, the photoassimilates are finally stored in the form reserved food material such as protein or oil.

**Table 2:** Year-wise data of grain yield from control and thiourea treated plants.

Treatments	Year (2010–11)	Year (2011–12)	Average (kg ha^−1^)
**Control**	1119±1	1210±15	1164
**Thiourea**	1238±7	1336±11	1287*

The grain yield was calculated at two successive years. The significance of mean difference (P<0.05) was evaluated on the basis of student t-test and marked with asterisk (*).

**Table:** Table 3. Measurement of quantum yield parameters at PSII and PSI in control and TU treated leaves.

Quantum yield parameters at PSI	Control	Thiourea
Effective quantum efficiency [Y(I)]	0.930±0.02	0.965*±0.04
Acceptor site limitation [Y(ND)]	0.029±0.01	0.017*±0.004
Donor site limitation Y(NA)	0.036±0.01	0.037±0.03
**Quantum yield parameters at PSII**		
Effective quantum efficiency [Y(II)]	0.582±0.03	0.669*±0.03
Non-light induced photochemical quenching [Y(NO)]	0.333±0.03	0.297*±0.02
Non-photochemical quenching [Y(NPQ)]	0.083±0.01	0.041*±0.01
Fv/Fm ratio	0.800±0.002	0.809±0.01

The values represent mean ± SD of five independent biological replicates. The significance of mean difference (P<0.05) was evaluated on the basis of student t-test and marked with asterisk (*).

In recent years, various transgenic based approaches have been tested to modulate source and sink strength; however, limited success has been achieved in the terms of increased crop yield [6], [14]. As an alternate strategy, the concept of strengthening the plant's *built in* mechanism using priming mediated physiological tuning, which does not involve any genetic modification, can be useful. The present study has proposed to use thiourea (TU, a known ROS scavenger [15]) for regulating source to sink relationship in plants. The rationale behind the selection of TU was based upon the fact that most of the steps for generating of photoassimilates at source and its translocation towards sink are regulated in a redox state dependent manner [16]. An additional support to this was derived from our previous findings, where the positive role of TU has been demonstrated for enhancing sucrose translocation [17] and for ameliorating salt [18], [19] as well as UV stress [20] in *Brassica juncea*. The outcome of this research not only highlights the significance of redox mediated regulation between source and sink, but also proposes the use of TU, as a bioregulatory agent, for enhancing crop productivity under field conditions.

## Results

### Thiourea treatment regulates the plant growth phenotype at different developmental stages

**Figure 1:**
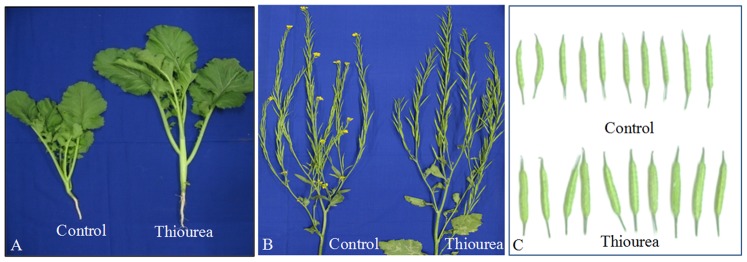
Differential phenotype of *Brassica juncea* plants at different states of growth. Differential phenotype in control and TU treated plants was observed at vegetative (A), flowering (B) and pod filling (C) stage. The experiment was repeated in two successive years to check reproducibility.

At the vegetative stage, the average shoot length and leaf area in TU treated plants were increased by 2.3- and 1.5-fold, respectively as compared to control (Fig. 1–A; Table-1). The growth observed at 65 DAS confirmed the onset of early maturity as the flowering stage was almost over in TU treated plants (Fig. 1–B). The significantly changed pod characteristics were also observed under TU treatment (Fig. 1–C). The yield attributes such as pod length, pod width, average number of seeds per pod, internodal distance between two pods and average weight for 1000 seeds were increased by 6, 35, 10, 20 and 3%, respectively in TU treated plants as compared to control (Table-1). The data obtained from two subsequent years confirmed 10% increase in crop yield under TU treatment as compared to control (Table-2).

### Enhanced photosynthetic efficiency under thiourea treatment

**Figure 2:**
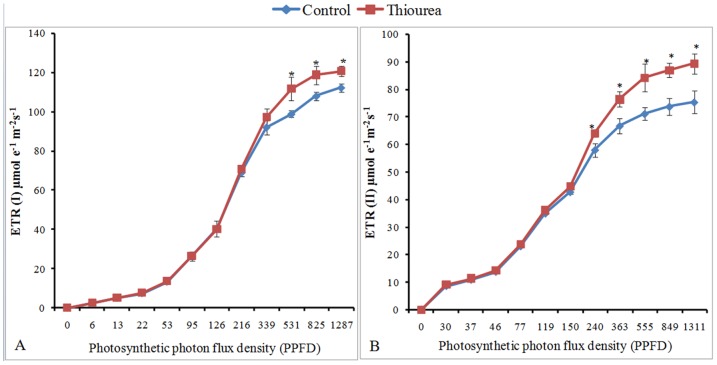
Electron transport rate (ETR) of PSI and PSII. The panel A and B represents the profile of ETR(I) and ETR(II), respectively in plants at 2 d after control and TU treatments. All measurements were performed over 6 cm^2^ area of fully expanded 2^nd^ leaf. The values represent mean ± SD of five independent biological replicates. The significance of mean difference (P<0.05) was evaluated on the basis of student t-test and marked with asterisk (*).

In response to TU treatment, effective quantum efficiency associated with PSI [Y(I)] and PSII [Y(II)] were increased by 4 and 13%, respectively, as compared to control. The increase at PSI was mainly due to the 41% decrease in donor side limitation [Y(ND)]. No significant change in acceptor side limitation [Y(NA)] was observed. At PSII, the TU mediated increase in YII was associated with 11 and 50% decrease in non-light induced photochemical quenching [Y(NO)] and non-photochemical quenching [Y(NPQ)], respectively over control. No significant difference between F_v_/F_m_ ratio was observed under control and TU treatment (Table-3). At higher photosynthetic photon flux density (PPFD), the TU treated leaves showed significantly higher electron transport rate at both PSI [ETR(I)] and PSII [ETR(II)], as compared to control (Fig. 2).

### Expression profiling of selected sugar transporters in source (leaves) and modulation in sucrose/starch ratio in source (leaves) and sink (pods)

**Figure 3:**
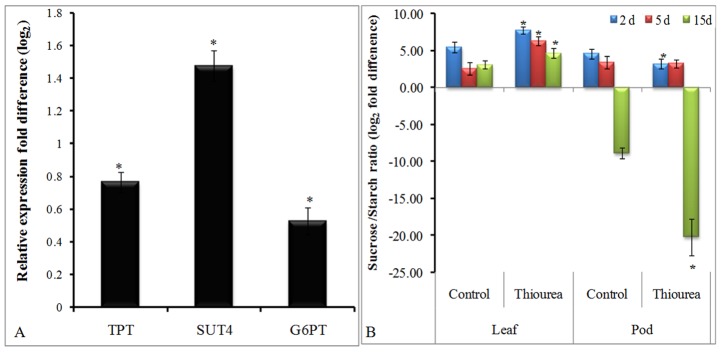
Expression profiling of sugar transporters in source and sucrose/starch ratio in source and sink. Panel-A represents the expression level of various genes such as *TPT, SUT-4* and *G6PT* in leaves at 2 d after control and TU treatment. The values represent the relative change in expression (log_2_ fold difference) in TU treated leaves as compared to that of control. The details of the gene-specific primers are mentioned in Table S1. Panel-B represents sucrose/starch ratio in leaf as well as pod of plants at 2, 5, and 15 d after control and TU treatment. The values represent mean ± SD of three independent biological replicates. The significance of mean difference (P<0.05) was evaluated on the basis of student t-test and marked with asterisk (*).

The expression level of major sugar transporter genes such as *TPT, SUT-4*, and *G6PT* was increased by 0.8-, 1.5- and 0.5-fold in leaves of TU treated plant as compared to control (Fig. 3–A). The sucrose to starch ratio in TU treated leaf was increased with the maximum of 3.8-fold at 5 d, as compared to control. This was in contrast to pod where the ratio was decreased at all the time points. The maximum reduction of 11.3-fold was seen at 15 d time point in TU treated pods as compared to control (Fig. 3–B).

### Modulation in the activities of enzymes determining source strength

**Figure 4:**
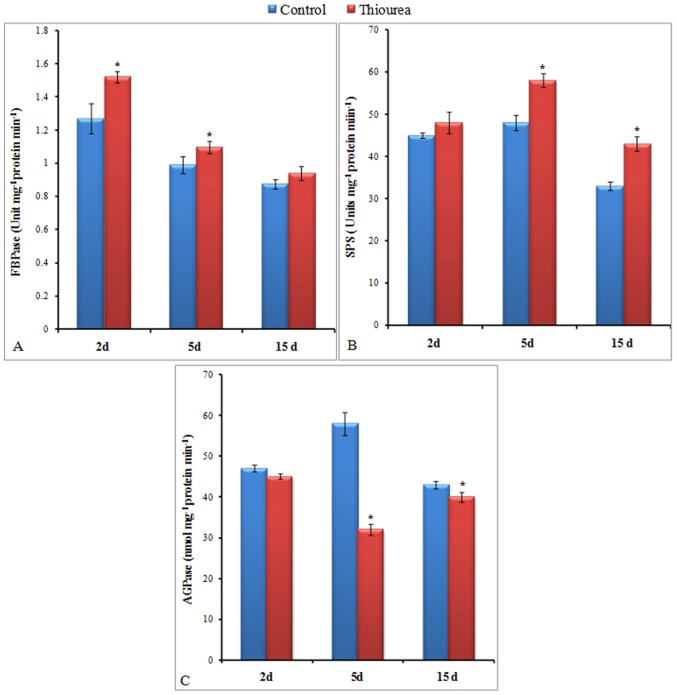
Activities enzyme determining source strength. The activities of fructose-1,6-bis-phosphatase (FBPase; A), sucrose phosphate synthase (SPS; B), ADP glucose pyrophosphorylase (AGPase; C) were measured in source leaves at 2, 5, and 15 d after control and TU treatment. The values represent mean ± SD of three independent biological replicates. The significance of mean difference (P<0.05) was evaluated on the basis of student t-test and marked with asterisk (*).

The activities of both FBPase and SPS in leaves were increased in response to TU treatment as compared to control. The maximum increase of 20% in FBPase activity was observed at 2 d time point and beyond that point, the extent of difference decreased continuously. At 15 d, no significant change in FBPase activity was observed between control and thiourea treatment (Fig. 4–A). This was in contrast with SPS activity that showed a time dependent increase of 6.7, 20.8 and 30.3% at 2, 5 and 15 d, respectively as compared to control (Fig. 4–B). Unlike FBPase and SPS, the activity of AGPase was decreased at all the time points. The maximum decrease of 45% was observed at 5 d in TU treatment as compared to control (Fig. 4–C).

### ^14^C-sucrose based radiotracer study to determine the direction of sucrose translocation

**Figure 5:**
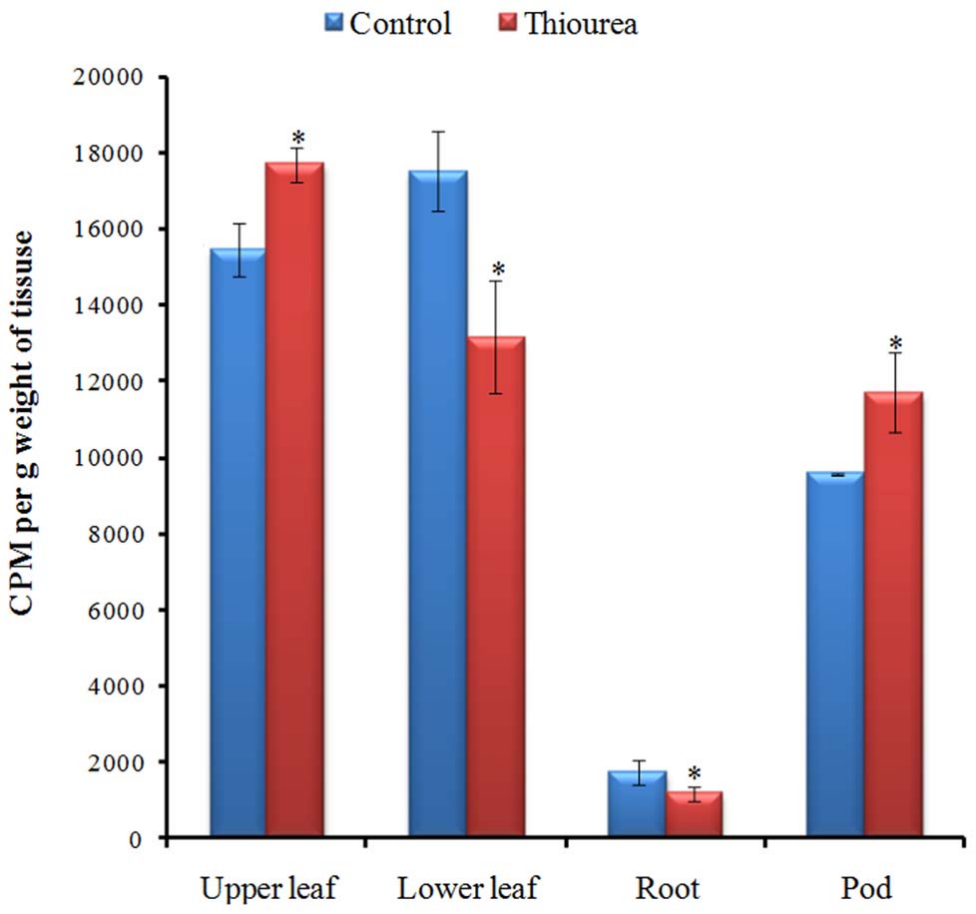
Measurement of sucrose translocation in different plant parts. The extent of sucrose translocation was measured using ^14^C-sucrose, as a radiotracer. The fixed quantity of ^14^C-sucrose was injected at the defined point in control and TU treatment. At 2 d after injection, immediate upper and lower leaf of injection, root and pod were harvested and ^14^C-sucrose level was quantified using scintillation counting. The values represent mean ± SD of three independent biological replicates. The significance of mean difference (P<0.05) was evaluated on the basis of student t-test and marked with asterisk (*).

The sucrose partitioning pattern in TU treated plants revealed the enhanced sucrose translocation towards pod. This was in contrast with control where a bidirectional movement of sucrose towards root as well as pod was observed. In pods, the level of ^14^C-sucrose was increased by 22% in TU treatment as compared to control (Fig. 5).

**Table:** **Table 4.** HPLC based quantification of different organic acids from control and TU treated pods.

Treatments		Phophoenol pyruvate (μg g-1)	Pyruvate (μg g-1)	Malate (μg g-1)
**2 d**	**Control**	29±0.4	22±0.6	494±40
	**Thiourea**	33*±0.6	22±0.5	499±18
**5 d**	**Control**	41±1.0	11±0.2	331±21
	**Thiourea**	50*±0.6	25*±2.0	468*±22
**15 d**	**Control**	29±1.5	7±0.4	Not detectable
	**Thiourea**	50±2.3	10*±0.6	466*±13

The values represent mean ± SD of three independent biological replicates. The significance of mean difference (P<0.05) was evaluated on the basis of student t-test and marked with asterisk (*).

### Modulation in the activities of enzymes for sucrose degradation in source and sink

**Figure 6:**
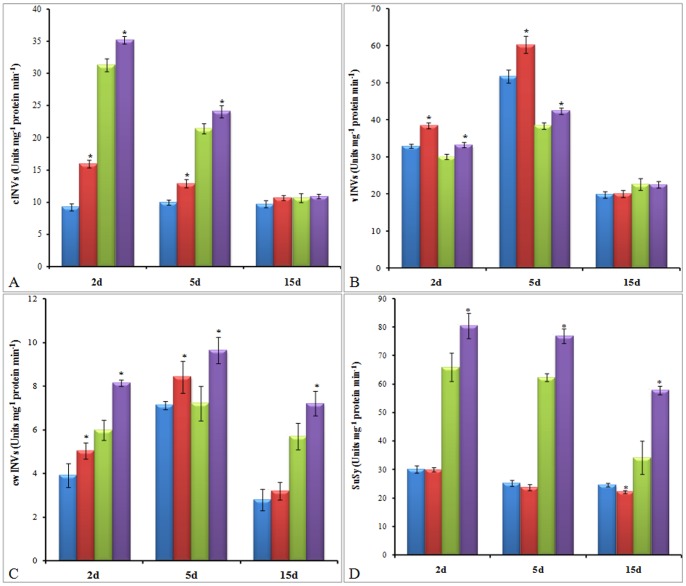
Activities of enzymes for sucrose degradation at source and sink. The activities of cytosolic (cINVs; A), vacuolar (vINVs; B) and cell wall bound (cwINVs; C) invertases and sucrose synthase (SuSy; D) were measured in leaves and pods at 2, 5, and 15 d after control and TU treatment. The values represent mean ± SD of three independent biological replicates. The significance of mean difference (P<0.05) was evaluated on the basis of student t-test and marked with asterisk (*).

In source as well as sink, the activities of all forms of invertases, such as cINVs, vINVs and cwINVs, were increased till 5 d of TU treatment (Fig. 6-A–C). However, at 15 d time point, no significant change was observed in either source or sink, except for cwINVs activity in pods which was 26% higher as compared to control (Fig. 6–C). At both 2 and 5 d time points, the order of invertases with respect to increased activity was cINVs> cwINVs> vINVs and cwINVs> cINVs> vINVs for leaves and pods, respectively. The maximum increase in the activity of cINVs (72%) and cwINVs (36%) was observed at 2 d after TU treatment in leaves and pods, respectively. In leaves, no significant difference in SuSy activity was observed at any time point, except for 15 d, at which it was decreased by 10% in TU treated leaves as compared to control. In TU treated pods, the SuSy activity was continuously increased by 22, 23 and 69% at 2, 5 and 15 d, respectively, as compared to control (Fig. 6–D).

### Modulation in the activities of enzymes for pod photosynthesis and oil biosynthesis at sink

**Figure 7:**
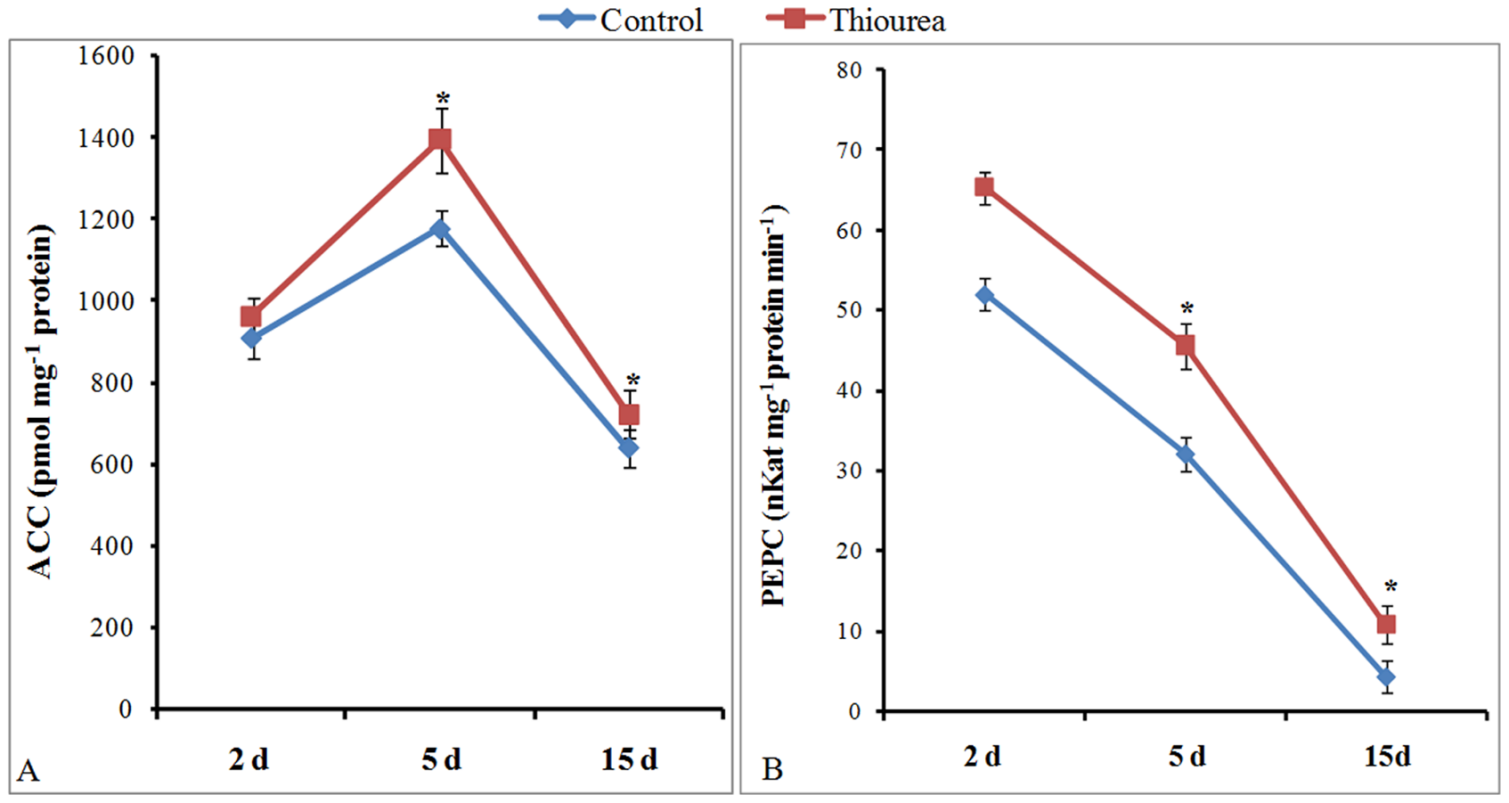
Activities of enzymes for pod photosynthesis and oil synthesis at sink. The activities of acetyl coA carboxylase (ACC; A) and phosphoenol pyruvate carboxylase (PEPC; B) were measured in pods at 2, 5, and 15 d after control and TU treatment. The values represent mean ± SD of three independent biological replicates. The significance of mean difference (P<0.05) was evaluated on the basis of student t-test and marked with asterisk (*).

In general, the ACC activity at all the time point was increased in TU treated pods as compared to control; however, the maximum of 15% was observed at 5 d time point (Fig. 7–A). The PEPC activity was also consistently increased under TU treatment as compared to control; however, it was associated with sharp time-dependent decline under both control and TU treated pods (Fig. 7–B).

### Effect of TU treatment on the level of organic acids [phophoenol pyruvate (PEP), pyruvate (PYR) and malate (MAL)] in pod

In general, the level of all the three organic acids was increased in response to TU treatment as compared to control; except for PYR and MAL for which no significant difference was observed at 2 d after treatment. The PEP level was increased in a time-dependent manner by 11, 18 and 42% at 2, 5 and 15 d, respectively as compared to control. The PYR level was increased by 56 and 33% at 5 and 15 d, respectively under TU treatment as compared to control. The MAL level was 29% increased at 5 d in TU treated pods as compared to control. No MAL level was detected in control samples at 15 d after treatment (Table-4).

### Effect of TU treatment on reserve food material

**Figure 8:**
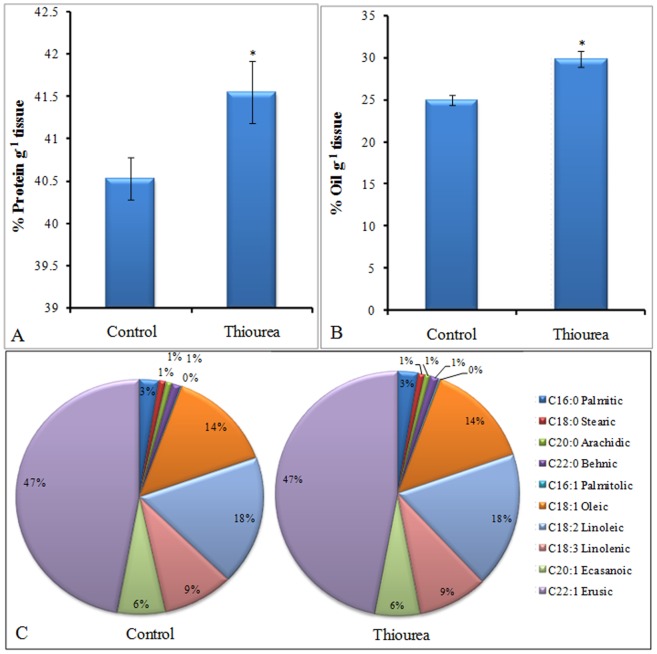
Status of reserved food material in seeds. The content of reserve food material such as protein (A) and oil (B) and profile of various fatty acids (C) was determined from the seeds derived from control and TU treated pods. The values represent mean ± SD of ten independent biological replicates. Each replicate represents the seeds pooled from 20 plants. The significance of mean difference (P<0.05) was evaluated on the basis of student t-test and marked with asterisk (*).

There was an increase in total protein (Fig. 8–A) and oil (Fig. 8–B) content by 2.5 and 19.2% respectively, in seeds derived from TU treated plants as compared to that of control seeds. Data obtained by GC-FID indicated that MUFA (Mono Unsaturated Fatty Acid), PUFA (Poly Unsaturated Fatty Acid) and SFA (Saturated Fatty Acid) remained unchanged in seeds derived from either control or TU treated plants (Fig. 8–C).

## Discussion

Thiourea (TU) is a non-physiological thiol and has been employed by various researchers to impart stress tolerance and improve yield of crops like mustard [21], wheat [22], mungbean [23], salt grass [24], potato [25] and maize [26]. The TU mediated enhanced yield was associated with an increased translocation of sucrose metabolites from source to sink [17]. Additionally, the molecular mechanism of TU mediated tolerance towards salt stress was also found to be associated with the maintenance of cellular redox homeostasis [19] and activation of various signaling and effector components of salt tolerance [18]. Towards this endeavor, the present study was undertaken to demonstrate the positive role of TU for enhancing the yield under natural field condition and to understand the underlying mechanism in Indian mustard (*Brassica juncea* L). At the vegetative stage, TU application improved the plant growth potential and photosynthetic efficiency. This was concomitant with the onset of early maturity and increased crop yield. All these effects could be attributed with TU ability to maintain redox homeostasis through its broad range of ROS scavenging activity which has been first demonstrated in HL 60 cell lines [15]. Later this has also been proved in plants by demonstrating its ameliorative action towards NaCl [19] and arsenic (Srivastava et al.; Unpublished) stress that are known to cause oxidative damage. The preliminary information about the mechanism of TU mediated action was derived in the terms of sucrose to starch ratio which is considered as an important indicator of source and sink strength [11]. In response to TU treatment, the ratio was consistently higher and lower at source and sink, respectively. This suggested the positive role of TU in orchestrating sugar dynamics and source-to-sink relationship of plant.

To get more mechanistic insight into the mechanism, the status of source and sink strength was evaluated in response to TU treatment. The source strength of any plant is mainly governed by the rate of sucrose biosynthesis in actively photosynthesizing leaf [27]. In addition to active surface area of leaf, the efficiency of both the photosystems, in terms of effective quantum yield and electron transport rate at high light intensity, was also significantly increased in response to TU treatment, suggesting an overall increase in net photosynthesis. Although, the exact mechanism was not explored, however, this might be either due to the avoidance of oxidative photo inhibition at PSII [28] or overall increase in protein biosynthesis which also demands reduced redox environment [29]. The enhanced photosynthetic efficiency was found to be coupled with increased expression of TPT and higher enzyme activity of FBPase that indicated the availability of sufficient building blocks for sucrose biosynthesis in TU treated leaves. The foliar application of TU will generate the reducing environment that might facilitate the intra- and/or intermolecular-reduction of dithiol bonds present in most of the Benson-Calvin cycle enzymes and FBPase leading to their maximum activation [16]. Apart from carbon metabolism, FPBase has been shown to be associated with TU mediated salt tolerance in seeds of Indian mustard [18]. Recently, FBPase has also been demonstrated as a suitable marker for drought tolerance in a diverse set of rice population [30]. Since, the natural field conditions, adopted for the present study, are always associated with multiple stress components; therefore, the increased FPBase activity might be responsible for enhanced photosynthesis, source strength and growth of plants under TU supplementation. This is also supported by the fact that transgenic Arabidopsis with simultaneous overexpression of TPT and cytosolic FBPase showed the increased photosynthetic carbon assimilation and growth under moderate and elevated light conditions, as compared to wild-type plants [31]. The higher FPBase activity will generate the F-6-P that might be directed either towards starch or sucrose synthesis catalyzed by AGPase or SPS, respectively. For starch biosynthesis, the F-6-P gets transported into chloroplast in the form of G-6-P. Although, a slight increase in the expression level of G6PT (G-6-P-transporter) was observed in leaves of TU treated plants; however, it was not co-ordinated with increased AGPase activity. The AGPase is the rate limiting enzyme of starch biosynthesis and is regulated in a redox dependent manner. The reducing environment activates the enzyme through NADP-thioredoxin reductase C (NTRC)-mediated monomerization [32]; however, its lower activity was observed under TU treatment. This clearly suggested a redox-independent mode of AGPase regulation in plants. Similar hypothesis has also been given previously where *ntrc* mutants have been shown to have comparable level of starch than wild-type [33]. The lower AGPase activity was simultaneous with higher SPS in TU treated leaves. The SPS is the major regulatory enzyme for sucrose synthesis and SPS null mutants of Arabidopsis were shown to accumulate very low level of sucrose in leaves [34]. Thus, all these data together indicated that metabolite flux was towards sucrose synthesis in TU treated leaves.

The sucrose synthesis at source should be coupled with its continuous transport towards sink to avoid any feed-back inhibition. Although, the root, older leaves and pod are considered as important sinks; however, ^14^C-sucrose based radiotracer study confirmed the selective translocation of sucrose toward pod in TU treated seedlings. Such a biased push of metabolites towards pod is considered as highly desirable and is responsible for increasing the crop harvest index [35]. The supplementation of TU has been shown to maintain mitochondrial ATPase activity and hence, accelerated the seed germination process that requires hypoxic condition [36]. Since, the molecular drivers of sink strength (at pod) also operate at low oxygen condition [14], the enhanced mitochondrial ATPase activity might be responsible for improved sink strength; however, this needs to be validated in future. An additional strategy to avoid the accumulation of sucrose at source is to sequester them inside the vacuole. This is mediated by a tonoplast localized sucrose transporter (SUT-4) [37]. The significant upregulation of SUT-4 in TU treated leaves will facilitate the feed-forward regulation of sucrose biosynthesis.

The source and sink relationship is also regulated by two sucrose hydrolyzing enzymes such as INVs and SuSy responsible for the generation of hexose sugar. Although, the levels of individual hexose sugars have not been tested, however, the overall increase in the activities of different INVs and SuSy indicated their enhanced levels in both leaves as well as pods of TU treated plants. The continuous generation of hexoses will avoid the feedback inhibition of sucrose biosynthesis and unloading at source and sink, respectively with an overall effect in the form of increased net carbon fixation. Out of the different INVs, the activity of cwINVs was maximally increased, at an early stage, in TU treated pods which signifies its relative significance for sucrose unloading. Recently, a direct correlation between cwINVs activity and extent of sucrose translocation has been demonstrated in tomato under heat stress [38]. Additionally, owing to their ability to modulate ROS levels [39], the hexoses may also participate in the production of signals for regulating cell cycle and cell divisions vital for the establishment of young sinks [40], [14]. The comparative analysis of SuSy activity under control and TU treatment suggested its major role as a determinant of sink strength. This could be because SuSy is more efficient under low oxygen condition [13]. The hypothesis was supported by the fact that highest increase in SuSy activity in TU treated pods was observed at seed maturity stage having least oxygen. Apart from improvement in source and sink strength, efficient loading and translocation of photoassimilates are also required for maximizing their level at sink. The significant increase in apoplastic loading volume (Fig. S1) and sucrose translocation [17], under TU treatment, together suggested that whole plant carbon partitioning is regulated in a redox dependent manner which was ultimately reflected in the form of significant increase in yield attributes such as pod density, pod length, pod width, seed number/pod and average weight of the seeds.

Inside the pod, unloaded sucrose gets converted into triose phosphate which finally gets converted into pyruvate (PYR) either through glycolytic pathway or phosphoenol pyruvate carboxylase (PEPC) mediated C_4_ pathway. The first step of PEPC pathway involves the carboxylation of PEP into oxaloacetate (OAA) which is then converted to malate (MAL). The MAL gets decarboxylated via NADP^+^ linked malic enzyme to PYR and CO_2_. The PYR formed, through either of the pathway, directed towards fatty acid synthesis through acetyl-CoA carboxylase (ACC). The additional carbon fixed through PEPC pathway is termed as pod or silique wall photosynthesis and is considered as important for regulating seed oil content in Brassica species [41]. The increased level of metabolites (PEP, MAL and PYR) and higher enzyme activity (PEPC) together indicated the efficient pod photosynthesis in TU treated pods, especially at initiation (2 d) and rapid grain filling (5 d) stage. This was also coherent with higher ACC activity required for maintaining the high rate of oil biosynthesis. Both PEPC [42] and ACC [16] are known to be redox regulated with maximum activity observed under reducing environment. This might be the reason behind their enhanced activity in TU treated pods. All these changes were ultimately reflected in the form of increased reserve food material (both oil and protein); however, basic composition of oil, in terms of MUFA, PUFA and SFA percentage, was not altered in response to TU treatment.

**Figure 9:**
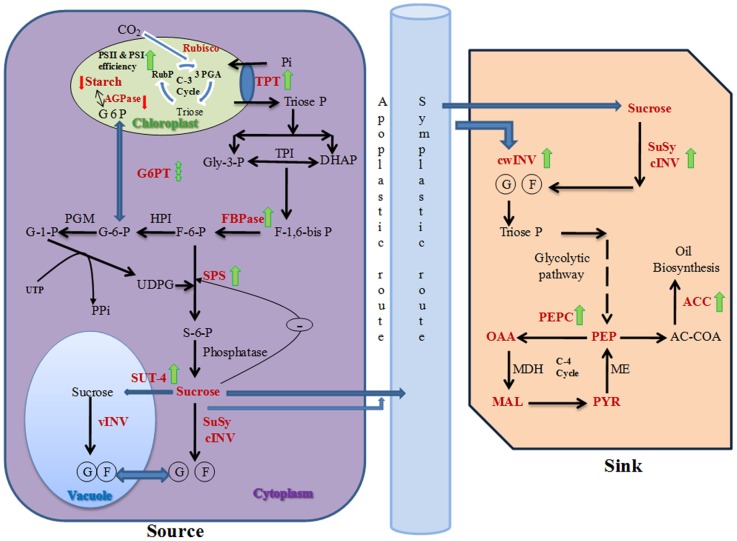
Working model for proposed mechanism of thiourea (TU) mediated action. Overall, the study deals with the modulation of source (leaves) and sink (pods) strength under TU treatment. The key steps are depicted as red bold letters. The green and red arrows represent the increase and decrease in expression/activity of associated gene/protein, respectively. The broken green arrow represents the non-coordinated change. The TU mediated increased photosynthetic efficiency coupled with higher expression level of sugar transporter (TPT) and activities of FPBase and SPS provided building blocks for enhanced sucrose biosynthesis at cytoplasm. The SUT-4 dependent vacuolar sequestration and enhanced activities of INVs and SuSy further supported the sucrose synthesis through feed-forward regulation. After unloading at sink, the photoassimilates gets channelized towards ACC-mediated oil biosynthesis either through glycolytic or PEPC pathway. Abbreviations: 3PGA: 3-phosphogyyceric acid; ACC: acetyl CO-A carboxylase; AGPase: ADP-glucose pyrophosphorylase; cINV: cytosolic invertases; cwINV: cell wall bound invertases; DAHP: dihydroxy acetone phosphate; F-6-P: fructose-6-phosphate; F-1,6-bis P: fructose-1,6-bis phosphate; FBPase: fructose-1,6-bis phosphatase; G-6-P: glucose-6-phosphate; G-1-P: glucose-1-phosphate; G6PT: glucose-6-phosphate transporter; Gly-3-P: glyceraldehyde-3-phosphate; HPI: hexose phosphate isomerase; MAL: malate; MDH: malate dehydrogenase; OAA: oxaloacetate; PEP: phosphoenol pyruvate; PEPC: phosphoenol pyruvate carboxylase; ME: Malic enzyme; PGM: phosphoglucomutase; Pi: inorganic phosphate; PYR: pyruvate; Rubisco: ribulose-1,5-bisphosphate carboxylase/oxygenase; S6P: sucrose-6-phosphate; SPS: sucrose phosphate synthase; SuSy: sucrose synthase; SUT: sucrose transporter; TPI: triose phosphate isomerase; TPT: triose-phosphate transporter; Triose-P: triose phosphate; UDPG: UDP-glucose; vINV: vacuolar invertases; glucose; fructose. 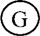 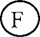

In conclusion, the study is based upon the concept of using TU (a non-physiological thiol based ROS scavenger) for modulating different plant processes such as source strength, translocation of photoassimilates, sink strength, pod photosynthesis and oil biosynthesis rate. A working model for TU mediated action has also been proposed (Fig. 9). Although, the associated key regulators were earlier known to be redox regulated, however, to the best of our knowledge, this is for the first time we have demonstrated that they can also be controlled by the external application of a redox molecule like TU with the ultimate effect in the form of increased crop yield and oil content. Considering the importance of *Brassica juncea*, the second most important edible oilseed crop after groundnut, which contributes to 27.8% for India's oilseed economy, there is a growing demand to enhance its production from its current production of 6.7 to 24 million ton by 2020 AD [43]. Towards meeting these criteria, strategies need to be put in place for enhancing productivity. The present study proposes to adopt the application of TU, as farmer's friendly technology, for enhancing the crop harvest index and oil content in Brassica.

## Materials and Methods

### Plant material, growth and treatment condition

The study was performed in Indian mustard (*Brassica juncea* L. var. TM-2). The surface sterilized seeds were distributed in two sets and then soaked separately with distilled water and thiourea (TU; 6.5 mM) for 6 h. Each set was sown in three different plots having an area of 9 m^2^ each. In total, there were six plots separated with an intermediate distance of 1 m. During the entire growth period (120 days; November to February), plants were fertilized twice with 90:40:60:40 kg ha^−1^ of NPKS fertilizer and watered thrice a week (for initial 2 weeks) followed by once a week till maturity. The set of plants derived from thiourea soaked seeds were also given two foliar applications of thiourea (250 g/ha) at 45 and 65 DAS (days after sowing) that represent the half inflorescence and early pod filling stage, respectively. In an earlier research, extensive field trials have been conducted to evaluate the efficacy of TU for enhancing yield of wheat and mustard crops under salt- and drought-affected lands of Rajasthan, India [21], [22]. These initial trails were used to select the optimal dose and mode of TU application for the present experiment. At 2, 5 and 15 d after the 2^nd^ foliar spray, 3^rd^ fully expanded leaf (source) and pod (sink) were harvested, frozen immediately in liquid nitrogen and then stored at −80°C until various biochemical analyses were performed. The selected time points represented three different stages such as early (2 d), rapid grain filling (5 d) and maturity phase (15 d). The harvesting time was fixed between 10:30–11 AM. The measurement of *in planta* photosynthetic efficiency and quantitative real-time RT-PCR analysis were performed only at 2 d after second foliar spray. The differential phenotype was recorded at vegetative (30 DAS; in terms of average shoot length and leaf area), flowering (70 DAS) and grain filling stage (100 DAS; in terms of pod length, pod width and inter-nodal distance recorded for 20 cm from shoot apex in the inflorescence). The yield parameters (average number of seed per pod and average weight of 1000 seeds), fatty acid profiling and quantification of oil and protein content were performed after seed harvest. The crop yield, in terms of seeds produced per ha of land, was calculated and the data obtained was revalidated in the subsequent year.

### Measurement of *in planta* photosynthetic efficiency

A dual-wavelength pulse-amplitude-modulated fluorescence monitoring system (Dual-PAM-100, Germany) was used for *in planta* determination of PSII and PSI efficiency. All measurements were carried out on 3^rd^ fully expanded leaf from shoot apex. The measurements were done over a leaf area of 6 cm^2^ of detached leaf. Before illumination, leaves were placed in moistened tissue paper for 20 min in dark. For PSII efficiency, minimal fluorescence after dark-adaptation (F_0_) was measured at low intensity beam light. A saturating pulse of 10,000 µmol photons m^−2^s^−1^ was then applied for 300 ms and then maximum fluorescence (Fm) was detected. At light adapted state, effective quantum efficiency [Y(II)], non-light induced photochemical quenching [Y(NO)] and non-photochemical quenching [Y(NPQ)] were calculated. For PSI efficiency, three parameters such as yield due to acceptor [(YNA)] and donor site [(YND)] limitation, and effective quantum efficiency [Y(I)] were calculated at saturated light impulse. For both PSII and PSI, an electron transport rate curve (ETR) has also been generated by exposing the leaves to increasing photosynthetic photon flux density (PPFD; at the rate of 1 min for each intensity) till steady-state is obtained.

### Quantitative real-time RT PCR analysis

All the primers used for Sybr green real time RT PCR were obtained from the *Arabidopsis thaliana* RT-PCR primer database [44].The detailed sequence of gene specific primers are mentioned in Table S1. The RNA isolation and quantitative real-time PCR were performed exactly as per the method described by [18].

### Measurement of sucrose and starch content

The freeze dried sample (100 mg) was extracted in 15 mL of 80% ethanol. The extract was boiled for 10 min and then centrifuged. The supernatant and pellet were used for the estimation of sucrose and starch using sucrose quantification kit (SCA-20; Sigma) and starch assay kit (STA-20; Sigma Aldrich), respectively as per the manufacturers protocol.

### Measurement of enzyme activities

The liquid nitrogen ground plant samples (∼500 mg) were homogenized in 1 mL of extraction buffer [Tris-HCl (100 mM; pH 8.0), MgCl_2_ (16 mM), EDTA (1 mM), dithiothreitol (20 mM), polyvinylpyrrolidone (2%; w/v), Triton X-100 (0.05%) and phenylmethylsulfonyl fluoride (2 mM)], squeezed through four layers of cheese cloth and then centrifuged at 12,000 *g* for 15 min at 4°C. A portion of the extract was used for the measurement of activities of different enzymes except for invertases for which the fractionation was performed. The specific methodology for estimation of enzyme activity has been given in supplementary methodology. The protein content in the sample was measured as per the method of Lowry et al. [45].

### Radiotracer studies using ^14^C-sucrose

Radio-tracer studies were performed on two independent set of plants grown in earthen pot containing 5 kg of soil. The irrigation, fertilizer application and thiourea treatments were similar to that of field plots. At the time of 2^nd^ foliar spray, uniformly labeled ^14^C-sucrose (2 µCi with 100 mM of carrier sucrose) was injected in the middle of third and forth fully expanded leaves using disposable syringe. For each plant, 80 µl was radioactive solution containing was added. At 2 d after injection, immediate upper and lower leaf of injection, root and pod were excised separately, weighed and then extracted in 10 mL of cold acetone. The extract (1 mL) was mixed with 5 mL of scintillation cocktail [naphthalene (30 g), PPO (2 g), ethylene glycol (100 mL), methanol (50 mL) were mixed and volume made up to 500 mL with dioxane] and then counted on protocol 2 of TRI-CARB 2100 TR liquid scintillation analyzer (Packard, Canberra). The efficiency of the counter used was 95%.

### HPLC based quantification of organic acids

For the quantification of different organic acids, samples (∼250 mg) were ground in liquid nitrogen and then extracted in 0.01 N H_2_SO_4_ (1:4 w/v basis). The content was centrifuged at 13,000 g for 10 min at 4°C and then supernatant was collected. All the samples were passed through 0.22 µ nylon syringe filters and then subjected for HPLC analyses. Separation and analysis of various organic acids was carried on reverse phase HPLC system (Waters, USA) with 10 µ C-18 analytical column (250 mm×4.6 mm). An isocratic elution was performed with milli-Q water (pH 2.1–2.15, adjusted with perchloric acid) at a constant flow rate of 1.0 mL min^−1^. Analytical grade organic acids (Sigma-Aldrich) were used as standards. The detection was performed at 210 nm using an UV detector. The chromatograms were recorded and analyzed using Empower software.

### Estimation of oil and protein content in seeds

For the estimation of oil content, 5 g of finely ground seeds from 10 independent plants was extracted in 250 mL of petroleum ether using repeated refluxing in soxhlet apparatus. After 6 h, the extract was transferred to a pre-weighed conical flask and subjected to vacuum evaporation (using Buchi rotavapor) till the constant weight of the flask was achieved. The oil percentage was calculated as per method described by Akbar et al. [46]. The protein content was estimated using Kjeldahl Analyzer (Kjeltec 2300 Analyzer unit, Foss Tecator AB, Hoganas, Sweden) as per the method described previously [47].

### Qualitative profiling of fatty acids

The seeds (0.2 g) were ground separately in 3 mL of petroleum ether, vortexed and then kept overnight for oil extraction. For each sample, ten independent biological replicates were prepared. Each replicate represents the seeds pooled from 20 plants. The fatty acid profiling was done using gas liquid chromatography as described previously by Mondal et al. [48].

### Statistical analysis

The experiment was laid out in a complete randomized design (CRD). The means were compared using Student's t test incorporated into Microsoft Excel 7.0 (Microsoft). Differences are described as significant (marked with *) when the P<0.05 was obtained.

## Supporting Information

Figure S1 **Measurement of apoplastic air (V_air_) and water (**V_water_ **) volumes from control and TU treated plants.** The V_air_ (A) and V_water_ (B) were measured in source leaves at 2 d after control and TU treatment. All measurements were performed over 4 cm^2^ area of fully expanded 2^nd^ leaf. The values represent mean ± SD of five independent biological replicates. The significance of mean difference (P<0.05) was evaluated on the basis of student t-test and marked with asterisk (*).
(DOC)

Methods S1 **Detailed methodology for the measurement of activities of various enzymes**.
(DOC)

Table S1 **Details of the primers used for quantitative real-time RT-PCR.** All the primers have been designed using the AtRTPrimer database (http://pombe.kaist.ac.kr/blan/genoPP.pl). Actin was used as a reference gene, allowing the gene expression values to be normalized.
(DOC)
