# Supplementary material for: Thiourea, a ROS Scavenger, Regulates Source-to-Sink Relationship to Enhance Crop Yield and Oil Content in Brassica juncea (L.)
Source: PLoS One. 2013 Sep 18;8(9):e73921. doi: 10.1371/journal.pone.0073921 (PMC3776803; doi:10.1371/journal.pone.0073921)
Supplement: Figure S1 — Measurement of apoplastic air (Vair) and water (Vwater ) volumes from control and TU treated plants. The Vair (A) and Vwater (B) were measured in source leaves at 2 d after control and TU treatment. All measurements were performed over 4 cm2 area of fully expanded 2nd leaf. The values represent mean ± SD of five independent biological replicates. The significance of mean difference (P<0.05) was evaluated on the basis of student t-test and marked with asterisk (*). (DOC) [file pone.0073921.s001.doc]

**Fig. S1: Measurement of apoplastic air (V_air_)** **and** **water (**V_water_**) volumes from control and TU treated plants.** The measurement of V_air_ and V_water_ was performed as per the method described by Husted and Schjoerring (1995). Briefly, leaf segments were infiltrated with high-viscosity silicone fluid (polydimethylsiloxane; viscosity 5 cs; Dow Corning, Poole, UK), blot dried with thin tissue paper and re-weighed. The V_air_ was calculated on the basis of increase in fresh weight. For V_water_, the leaf segments (4 cm^2^) were cut from leaves, washed thoroughly, blot dried and weighed. The leaf segment was infiltrated with indigo carmine dye (indigo-5,5'-disulfonic acid; 50 µM prepared in 50 mM phosphate buffer pH-6.2) and then quantification of V_water_ was performed on the basis of dye dilution. The data obtained showed that V_air_ (A) and V_water_ (B) were increased by 55 and 80%, respectively, in response to TU treatment as compared to control.

**References**

Husted S, Schjoerring JK (1995) Apoplastic pH and ammonium concentration in leaves of Brassica napus L. Plant Physiol 109: 1453–1460.
