# Supplementary material for: Thiourea, a ROS Scavenger, Regulates Source-to-Sink Relationship to Enhance Crop Yield and Oil Content in Brassica juncea (L.)
Source: PLoS One. 2013 Sep 18;8(9):e73921. doi: 10.1371/journal.pone.0073921 (PMC3776803; doi:10.1371/journal.pone.0073921)
Supplement: Methods S1 — Detailed methodology for the measurement of activities of various enzymes. (DOC) [file pone.0073921.s002.doc]

**Supplementary methods: Detailed methodology for the measurement of activities of various enzymes.**

***Fructose-1,6-bis-phosphatase (FBPase; EC 3.1.3.11)***

The FBPase activity was determined by measuring the rate of NADP to NADPH reduction at 340 nm. The reaction mixture contained TRIS-HCl (100 mM; pH-8.8), fructose-1,6-bisphosphate (0.6 mM), Na-EDTA (0.5 mM), MgCl_2_ (10 mM), NADP^+^ (0.3mM), glucose-6-P-dehydrogenase (0.6 units), phosphoglucose isomerase (1.24 units) and enzyme extract. A blank reaction was run for each extract where fructose 1-6 phosphate was absent. The activity was given in terms of units mg^-1^ protein (1 unit was considered equivalent to 1 µmol of NADPH produced min-¹).

***Sucrose phosphate synthetase* (*SPS; EC 2.4.1.14*)**

The SPS activity was determined by measuring the UDP level through pyruvate kinase (PK)/lactate dehydrogenase (LDH) coupled reaction. The reaction mix contained HEPES/NaOH (100 mM; pH 7.5), glucose-6-phosphate (25 mM), fructose-6-phosphate (5 mM) and UDPG (20 mM). The reaction was carried at 30°C for 45min and then terminated by boiling at 100°C for 3 min. The UDP formed was quantified by rate of NADH oxidation using PK/LDH mix (450 U each) in TRIS/HCl buffer (100 mM; pH-7.5), phosphoenolpyruvate (0.8 mM) and NADH (0.3 mM). Control samples were first boiled at 100ºC for 4 min. The absorbance was taken at 340 nm and one unit was expressed in terms of 1 nmol of NADH consumed per minute per mg of protein.

***ADP glucose pyrophosphorylase* (*AGPase; EC 2.7.7.27*)**

The activity of ADPG was assayed by nonradioactive endpoint assay. The amount of G-1-P produced was estimated by coupling with NADH production using phosphoglucomutase and G-6-P dehydrogenase. The reaction mix and blank was prepared as per the method described in Vu et al. 2001.

***Cytosolic (cINVs), cell wall bound (cwINVs) and acid-soluble vacuolar (vINVs)*** ***invertases***

The sample (250 mg) was ground to fine powder in liquid nitrogen and then extracted in1 mL of homogenization buffer containing HEPES-KOH (50 mM; pH 7.5), EDTA (1 mM), EGTA (1 mM), benzamidine (2 mM), DTT (5 mM), and phenylmethylsufonyl fluoride (0.1 mM). The homogenate was centrifuged for 30 min at 20,000g and the supernatant was used for the measurement of cINVs and vINVs. The pellet was washed four times with demineralized H_2_O and then incubated overnight in 1 mL of homogenization buffer containing 1M NaCl. The released enzymes was recovered by centrifugation and then used for the measurement of cwINVs. For cwINVs and cINVs, the reaction mix containing HEPES-NaOH (50 mm; pH-7.5), sucrose (0.5 m) and enzyme extract was incubated at 37ºC for 30 min and then stopped by boiling for 5 min. The released hexose sugar was estimated using DNSA reagent and then used for estimating the invertases activity. For vINVs, the reaction mix contained sodium acetate (250 mm; pH-4.5), sucrose (0.5 m) and enzyme extract. The reaction was stopped by the addition of 100 µl of ZnSO_4_ (50 g L^−1^) followed by 5 min incubation on ice and 5 min of boiling. The zinc was then precipitated by addition of 400 µl of K_2_CO_3_ (100 mm) and released hexoses were estimated using dinitrosalicylic acid (DNSA) reagent as per the protocol given in Miller et al (1959).

***Sucrose synthase (SuSy; EC 2.4.1.13)***

The SuSy activity was measured in cleavage direction by coupling the formation of UDP-glucose with the reduction of NAD in the presence of UDP-glucose dehydrogenase. The reaction mixture contained HEPES-NaOH buffer (20 mM; pH-7.5), sucrose (100 mM), UDP (2 mM), MgCl_2_ (2 mM), UDP glucose dehydrogenase (0.005 U), NAD^+^ (1.5 mM) and enzyme extract. Sucrose synthase activity was calculated in terms of µmol NAD^+^ reduced per min per mg protein during UDP glucose dehydrogenase mediated conversion of UDP-glucose to glucose and UTP. A blank reaction was run for each extract where sucrose was absent.

***Acetyl coA carboxylase (ACC; EC 6.4.1.2)***

The ACC activity was determined by measuring the incorporation of ^14^C from NaH^14^CO_3_ into malonyl-CoA. The reaction mix contained N,N-bis [2-hydroxyethyl]glycine (Bicine)-NaOH (60 mM; pH 8.0), ATP (6 mM), MgCl_2_ (5 mM), 6 mM NaH^14^CO_3_ (23 M Bq/mmol) and acetyl-CoA (0.7 mM). The reaction was started by adding 40 µl of extract. After 12 min of incubation at 37 ºC, the reaction was stopped by the addition of 50 µl 6 mM HCl. A portion of reaction mix was taken into scintillation vial, dried at 40°C and then used for liquid scintillation counting as per the method described above for ^14^C-sucrose. The ACC activity was expressed in the terms of H^14^CO3 incorporation into acid-stable compound.

***Phosphoenol pyruvate carboxylase (PEPC; EC 4.1.1.31)***

The PEPC activity was assayed by monitoring the formation of oxaloacetate in a malate dehydrogenase coupled reaction. The reaction mix contained HEPES-KOH (50 mm; pH-8), MgCl_2_ (5 mM), PEP (2.5 mM), NADH (0.15 mM), NaHCO_3_ (2 mM) 15% (v/v) glycerol and malate dehydrogenase (5 units/mL). The PEP-dependent NADH oxidation was monitored at 340 nm. A blank reaction was run for each extract where PEP was absent. One unit of activity defined as one µmole of NADH oxidation per minute at 25°C.

**References**

Miller GL (1959) Use of dinitrosalicylic acid reagent for determination of reducing sugar. Anal Chem. 31: 426.

Vu JCV, Gesch RW, Pennanen AH, Allen Jr LH, Boote KJ, Bowes G (2001) Soybean photosynthesis, rubisco, and carbohydrate enzymes function at supraoptimal temperatures in elevated CO_2_. J Plant Physiol 158: 295–307.
