# Supplementary material for: Thiourea, a ROS Scavenger, Regulates Source-to-Sink Relationship to Enhance Crop Yield and Oil Content in Brassica juncea (L.)
Source: PLoS One. 2013 Sep 18;8(9):e73921. doi: 10.1371/journal.pone.0073921 (PMC3776803; doi:10.1371/journal.pone.0073921)
Supplement: Table S1 — Details of the primers used for quantitative real-time RT-PCR. All the primers have been designed using the AtRTPrimer database (http://pombe.kaist.ac.kr/blan/genoPP.pl). Actin was used as a reference gene, allowing the gene expression values to be normalized. (DOC) [file pone.0073921.s003.doc]

**Table S1: Details of the primers used for quantitative real-time RT-PCR.** All the primers have been designed using the AtRTPrimer database (http://pombe.kaist.ac.kr/blan/genoPP.pl). Actin was used as a reference gene, allowing the gene expression values to be normalized.

| SUT-4 | For-cgctcgttgggcatagtagcgatag |
| --- | --- |
|  | Rev- ccgctgagacgcttaggatcgtagt |
| TPT-1 | For-gtccgtgggtcttcctaaacgtg |
|  | Rev-ccgagccagttgaatgatagctcag |
| G6PT | For-tttgggtgaaaccttccctacttcg |
|  | Rev-cccacatctgaggtccttcaacag |
| Actin | For- ctcctgccatgtatgtcgctatcc |
|  | Rev- aaggtccaaacgcagaatagcatgt |
